# Ageing, Disability, and Spinal Cord Injury: Some Issues of Analysis

**DOI:** 10.1155/2018/4017858

**Published:** 2018-11-19

**Authors:** Roberto Pili, Luca Gaviano, Lorenzo Pili, Donatella Rita Petretto

**Affiliations:** ^1^Comunità Mondiale della Longevità, Worldwide Community on Longevity, Italy; ^2^Department of Education Psychology and Philosophy, State University of Cagliari, Italy

## Abstract

Spinal cord injury is a disabling disorder, worldwide spread, with important consequences on functioning and health conditions and impacts on physical, psychological, and social well-being. The consequences are related to the lesion itself and to other complications related to the lesion. In the last decades, there have been an increasing of the mean ages of onset and also an increase in life expectancy after the lesion. So, differently from the past, people with spinal cord injury can age after the lesion. Taking into account the need to share data and information about specific disabling conditions and their relationship with ageing, this paper aims to discuss some issues from recent literature on the relationship between aging and disability in the spinal cord injury, according to a narrative review approach. A narrative review of the literature on ageing and spinal cord injury was undertaken. Search was based on the following electronic databases: PubMed/Medline and Ovid/PsychINFO. A combination of the following keywords was used: (1) “ageing” or “aging” and (2) “spinal cord injury” or “spinal cord lesion” and (3) disability. Data on consequences of the lesion in the life of aging people, secondary health conditions, life expectancy, participation, and quality of life are discussed. Then, a brief discussion of clinical issues and the role of interventions aimed to promote wellbeing, health, quality of life, and participation of people with spinal cord injury is proposed.

## 1. Introduction

Ageing is a complex phenomenon, related to genetics, constitutional variables, lifestyles, and environmental variables [1, 2]. During the human life, there are different phases of development: in the first phases of life, there is a progressive increasing of functioning (from infancy to adolescence), there is a sort of plateau during adult life, and then there is a physiological reduction of functioning in ageing. The speediness and the quantity and quality of this reduction are related to genetic variables (about 25%), but most of all they are related to lifestyles and environmental variables (about 75%) [1, 3–7]. A progressive reduction of functioning (related to genetics, to constitutional variables and to lifestyles), together with negative environmental factors, could lead to diseases, to disorders, to functional limitations, and to disability.

Nowadays, according to the vision proposed by International Classification of Functioning, disability and Health of the World Health Organization [8], and other conceptual models of disability and approved by the United Convention of the Rights of the People with Disabilities [9, 10], disability is viewed in a dynamic way and as a process. According to this vision, disability is the consequence of the relationship of the person, with his/her health conditions, and the environment [8–10]. There is also an international agreement in the view that “health and active ageing” is not without disorders or without diseases, but it refers to wellbeing from a biopsychosocial point of view: so it refers to wellbeing and quality of life, even in the presence of a disease or a disorder [11–15]. These topics are strictly subjective and individually and socioculturally defined [11–15]. From the more recent conceptual models of ageing and disability, the aim of each kind of intervention is to prevent pathological ageing, to reduce the risk of age-related health conditions and their consequences, to promote active and health ageing, and to prevent the changing from usual to pathological ageing [11–15]. From both the conceptualization of ageing and the conceptualization of disability, the main feature and the key element that make a good quality of life is, first, the recognition of the centrality of the person; second, it is the possibility to maintain, along all the phases of life, autonomy (that means maintain control and decision making in the different domains of ourselves life), independency (the ability to choose and do, also with help, activities of daily living), and a good quality of life [8–10, 12–15].

Worldwide, there is an increasing number of people with disability and people aging with disability. In these years there have been two different processes, strongly correlated with each other. On one side, there is worldwide progressive aging of population, the so-called “demographic revolution” or “demographic transition,” by which there is an increasing of general life expectancy. On the other side, thanks to the increasing number of quality of sociosanitary services and sanitary services, there is also an increasing of life expectancy for people with disability, so they can age with previous health disabling conditions. According to the report on disability published by World Health Organization and the World Bank [16], there is an estimation that about 15.3% of people were people with disability in 2004 and about 15% of people in 2010, with about 2-4% of these people with disability, have severe functional limitations. There is an important effect of age: the higher the age, the higher the frequency of disability [16]. For these reasons, the relationship between aging and disability has become a very important one for its consequences on participation, inclusion and quality of life of ageing people and for its consequences on socio-sanitary organizations [17–21]. In 2002, Verbrugge and Yang described two kind of relationship between disability and ageing: “ageing with disability,” which refers to people with disability that can age with previous health disabling conditions and thanks to the increasing of the quality of sociosanitary services and sanitary services now have an increasing of life expectancy for people with disability, so they can age and live more years; and “disability with ageing,” which refer to ageing people that become people with disability only during his/her ageing process, mainly due to age-related conditions [17]. Some years later, some authors felt the need to create a bridge between the two ways and have a look to the close relationship between the two different kinds of relationships described earlier, taking into account the similarities between them, even if the onset of the disabling conditions could be in different phases of life [19, 20].

Aiming to clarify further these aspects, Campbell and Putnam [21] described three kinds of relationships or consequences between ageing and disability. A first kind of consequences is “disability-related secondary conditions”: people with disability can have an increased likelihood of secondary conditions directly or indirectly (any additional physical or mental health conditions that could results from a primary disabling conditions, but are not a specific feature of it), similar to those experienced by ageing people in general, but they occur about 20-25 years sooner, and they are often described as premature, atypical, and having accelerated ageing [21]. A second kind of consequences is “age-related conditions” that could be experienced by aging people and also by ageing people with disability; these conditions are related to the aging and to the long-term effects exposure to environmental hazards, or to the effects of poor health behaviors. Some examples are hypertension, high cholesterol, diabetes, osteoarthritis, heart disease, gait and mobility problems, falls, respiratory infections/chronic obstructive pulmonary disease, urinary incontinence, osteoporosis, skin disease, hearing and vision loss, and dementia. A third kind of consequences is “Multiple Chronic Conditions,” the risk to have two or more different chronic conditions together, in dyads (hypertension and diabetes), or in triads (cholesterol, hypertension, and diabetes). According to Campbell and Putnam [21], these three kinds of relationships between ageing and disability are very closely related to one another and they have clear influences on health, quality of life, daily life, participation for ageing people and social costs, and subjective and objective burden for family and relatives. The authors claimed that the three kinds of consequences could happen in people with disability or ageing people, with qualitative and quantitative differences, but also with similarities: persons aging with disability and older adults share a set of chronic conditions, both as a disability related secondary conditions and as age-related chronic conditions. Moreover, people with disability could experience also age-related chronic conditions and disability-related secondary conditions and ageing people and people with disability can have multiple chronic conditions. So, the similarity between the two groups is more than the differences and an approach that does not take into account these aspects could have the risk of not promoting health and wellbeing of people. Aiming to overcome these risks, the authors proposed to close the gap and create a bridge between ageing with disability and disability with ageing (and also between ageing and disability) and to focus attention to health promotion programs aimed to reduce the burden of chronic conditions of people ageing with disability. Most of all, the authors highlight the importance to study the relationship between aging and disability, according the proposed approach, and the need to study and share information about these field of study and to share data and information about specific disabling conditions [21].

Spinal cord injury is a disabling disorder, worldwide spread, with important consequences on functioning and health conditions and impacts on physical, psychological, and social well-being, due to the lesion itself and to other complications related to the lesion [22, 23]. Taking into account these two sides of the relationship between ageing and disability and the proposal of Campbell and Putnam [21] to share data and information about specific disabling conditions, this paper aims to discuss some issues from recent literature on the relationship between aging and disability in a specific disabling condition, the spinal cord Injury, according to a narrative review approach. We will also discuss clinical issues and the role of interventions aimed to promote wellbeing, health, quality of life and participation of people with spinal cord injury. A narrative review of the literature on ageing and spinal cord injury was undertaken. Search was based on the following electronic databases: PubMed/Medline and Ovid/PsychINFO. A combination of the following keywords was used: (1) “ageing” or “aging” and (2) “spinal cord injury” or “spinal cord lesion” and (3) “disability”. Only studies published in English were included. The reference list of the retrieved reviews was examined, too, to identify potential additional studies. Articles that do not have a clear focus on spinal cord injury and ageing were discarded. Search criteria were as follows: two authors (LG and RP) assessed all the retrieved articles for inclusion, on the basis of their titles and abstracts, according the following criteria: (1) written in English (2) had a participant or group of participants identified as aging persons with spinal cord injury. The selected articles were then reviewed by two more authors independently and the same authors inspected the full texts (DRP and LP). Due to the fragmented nature of the selected articles, the different methodologies used and the qualitative differences of the studies, only a narrative analysis of the data in the selected articles was made. In the following paragraphs a narrative description of the results derived from the papers included.

## 2. Spinal Cord Injury and Ageing

Because full neurologic recovery is very difficult to achieve after the lesion, the spinal cord injury has long-term consequences on functioning and quality of life [22, 23]. In a spinal cord injury, the structures and function of the spinal cord are damaged by trauma, inflammation, tumors, or other causes, resulting in impairment of motor, sensory and autonomic function below the level of injury [2–23]. In general, in the short term, the effects of the lesions on the functioning of the person depend on the level of lesion, the quality of the lesion (complete/incomplete), and the causes of the lesion. The higher the lesion in the spinal cord, the more serious the effects and the higher the risks of mortality if the lesion impairs the respiratory system [2]. But different variables could mediate the results of the lesion on long-term period and on the quality of life, participation, and inclusion of people with spinal cord lesion. There can be two main kinds of spinal cord injury: traumatic lesion (mainly related to traffic accidents, falls, domestic accidents, violence, and other causes) and not-traumatic lesion (due to tumors, infections, and other disorders). The traumatic one is the more frequent situation [2, 23]. According to different studies, the annual incidence varies from to 16 to 54 new cases per million population, with a mean of 23 new cases per million population and with great differences between countries [2, 22–29].

The relationship between ageing and spinal cord injury has two sides: the increasing of the mean age of onset and the increasing of life expectancy after the lesion [2]. Prior to World War II, the life expectancy after spinal cord injury was very low, and people with spinal cord injury were not likely to survive for a long time after the lesion [30–32]. In the early 1970s, the improvement of knowledge and treatment about acute treatment and rehabilitation led to a decrease in premature mortality [30]. During the last decades the life expectancy for person with spinal cord lesion has increased again, thanks to the increasing of medical and rehabilitation knowledge (the topic of increasing life expectancy after spinal cord injury is still on debate, with contrasting data. Starting from the First World War there has been an increase in life expectancy for people with spinal cord lesion, but there has been a sort of plateau during 1980s and now it is still lower than in noninjured populations. Moreover, there is a greater rate of mortality among people with severe SCI (more if there are pulmonary and respiratory complications related to the level of injury) [2].) [33]. So, differently from the past, people with spinal cord injury can age after the lesion.

The second side of the relationship between ageing and spinal cord injury is the increasing of the mean age of the lesion, correlated with the worldwide progressive ageing of population. There is an increase in the number of persons and new causes of lesion later in life, like falls for traumatic lesions and degenerative disorders for nontraumatic lesion [26–29, 34]. According to Groah and colleague [34], the mean age at the time of injury in the United States has increased of about ten years in the period 1970 to 2002 (from 28 years to 38 years) and it was about 40 years during the 2005-2009 period. Frontera and Mollett [2] reported an increase of average time of injury from to 42 years in 1970 to 42 years in 2010. Molton and Yorkston [35] reported that the 30% or 40% of people with spinal cord injury in western countries are over 65 years old, but also in eastern countries, the proportion of middle or elder age groups at injury is increasing [29]. According to Ge and colleague [23] the increase of mean age is related to the risks of falls in over 65 years old population. However, with reference to different epidemiological studies on the relationship between age on onset and spinal cord injury, there is a bimodal age-of onset distribution: there exist two different groups of ages, one younger where the main causes are traffic accidents and one older where the main causes are falls and other kinds of accidents (domestic accidents) [36].

Sing and colleagues [22] claimed that the increase of age of onset is also related to the increasing of nottraumatic spinal injury and the presences of previous comorbidities that affects general outcomes and risks of mortality.

So, in the last decades an increasing number of older people has spinal cord injury, both with spinal cord lesion early in their life or late in their life, with the onset of the lesion in different phases of life. Various authors highlighted the need to take into account these epidemiological change and now we have the possibility to consider it also thanks to longitudinal studies and epidemiological studies [2, 22, 23, 27–29, 33, 37].

## 3. Consequences and Causes of Spinal Cord Injury in Ageing: A Complex Relationship

According to those previously described aspects, there are people with spinal cord lesion that became older after the lesion and there are people that meet the lesion in their aging process. From this point of view, the relationship between aging and spinal cord injury is a very complex one. In 2010, Yorkston and colleague [38] tried to describe the relationship between ageing and spinal cord injury describing the perception of people with spinal cord injury and other disabling conditions. In a qualitative analysis of the themes proposed by people, they found five major themes: identity, progression of physical symptoms, psychosocial pathways of adaptation, the variation of health care services, and concerns about the future, related to the changing in the disorders and in comorbidities. Based on their findings, the authors described multiple pathways, some positive and favorable (like adjustment and medical advancements) and some negative and not favorable (like physical decline and concerns about the future). They also claimed that while some pathways have a progressive course, like physical decline and the onset of comorbidities or other causes of functional decline related to the lesion itself or its secondary consequences (for example, overuse of body part for mobility, leading to other problems like shoulder disorders), other pathways are not so strictly progressive and are less studied and understood. The authors claimed that quality of life and adjustment to illness are not well studied and understood and more studies are needed [38].

An aspect of the relationship between ageing and spinal cord injury is a sort of “accelerated ageing” of organ systems [2, 27, 36, 39]. Since 1992, DeVivo and colleagues [39] highlighted also the role of age of onset: the higher the age of onset, the higher the effects on functional decline. For example they found that individuals older than 61 years were more like to develop some kinds of serious secondary conditions immediately after the lesion (like pneumonia, gastrointestinal hemorrhage, pulmonary emboli, or renal disorders) and the age of onset could have other effects on re-hospitalization and long-term consequences. In recent years, also Hitzig and colleague highlighted also the role of age of onset: the higher the age of onset, the higher the effects on functional decline and these effects are amplified by the presence of pre-existing co-morbidities that further affect outcomes [36].

Frontera and Mollett (2017) [2] analyzed the relationship between aging and spinal cord injury, describing this addictive effect of age and they propose an explanation. Generally, from 25 years of age, there is a reduction of functional capacity of about 1% per year. In spinal cord injury there is an acceleration of functional and metabolic decline, higher during the first period immediately after the event. Rodakowski and colleagues [40] reported an accelerated aging in older with spinal cord injury, based on an increased frequency of secondary health complications and an increased risk of re-hospitalizations. The effects on autonomy and activities of daily living are not only directly linked to age but also to other factors, for example, like participation and living in the community limited the effects on activities of daily living. Based on their data, the authors proposed to consider them in the development of intervention aimed to “buffer” the effects of accelerated aging in spinal cord injury [40].

There are some peculiar causes in spinal cord injury in older people. Chen and colleagues [28] reported that among the elderly slipping, tripping, stumbling, and falls constituted the most common etiology, accounting for 60% of all SCIs. Transport accidents were the second leading cause of SCI among the elderly (24%), followed by complications of medical and surgical care (12%). There are also qualitative differences in the same causes: for example, falls in older are generally low energy falls, from lower height, and they produce less severe incomplete injury, while in younger are generally from higher height and they could produce a more severe injury [41].

In general, in ageing with spinal cord injury there can be all the three consequences described by Campbell and Putnam [21]: age-related conditions, accelerated ageing and multiple chronic conditions. According to Frontera and Mollett [2] the evidence of multiple complications in ageing with spinal cord injury is related both to increase of the age of onset and to the ageing of people with previous spinal cord injury. The main co-morbidities are neurologic diseases (spasticity, weakness, pain and neurogenic blower and bladder), osteoporosis and other musculoskeletal disorders (like bone mineral density (BMD) loss), cardiovascular diseases (an accelerated risks for cardiovascular disorders, the so-called “Cardio metabolic syndrome” that includes insulin resistance, obesity, and hypertension), metabolic syndromes and mental health disorders (mainly depressive disorders) [2, 42, 43]. There different estimates of depressive disorders in people with spinal cord injury: about 1 in 5 persons are diagnosed with depressive disorders, even in there are different data in different studies. According to Bombardier and colleagues [44], there is an high risk that depression could be under-detected and not treated in people with spinal cord injury, due to the difficulty in distinguish somatic complaints of depression from secondary symptoms of the lesions (like pain and fatigue for example).

Molton and colleagues [35] highlighted the need to distinguish between primary consequences of spinal cord injury and secondary consequences that are indirectly influenced by the lesion. These secondary health conditions include chronic pain, fatigue, balance problems or worsening of spasticity, immunosuppression, urinary tract infection and pressure ulcers. According to Molton and colleagues [35], it is important to make this distinction because the secondary health conditions could have very important effects on functioning and participations and they can be prevented or controlled. The authors also claimed that the secondary health conditions and the chronic conditions (like hypertension and diabetes) are more frequent in older age, while in younger ages there are more psychosocial and somatic concerns [35].

Jorgesen and colleagues [26–37] claimed that it is important to distinguish between secondary health condition in spinal cord injury and those that are more frequent in older with spinal cord injury: the most common secondary health conditions in spinal cord injury are pain, neurogenic bowel and bladder, fatigue, osteoporosis. Some secondary health conditions are more frequent in older individual with spinal cord injury or those with longer period from the lesion, like cardiovascular disease, diabetes, bone mineral density loss, fatigue and respiratory complications or infections.

Ahn and colleagues [41] highlighted that aging could be also related to different approach in acute treatment and rehabilitation. They reported that people with traumatic spinal cord injury who were aged 70 years or older who had less severe incomplete neurologic injuries also experienced delays in transfer to a specialized treatment center and further delays from admission to surgery and they experience significantly higher rates of adverse events and had higher mortality [41]. More studies are needed on these aspects.

In the last decades, some authors have analyzed the effects on quality of life and participation of spinal cord injury itself, secondary health conditions and other chronic conditions age-related in ageing people with spinal cord injury. For example, Jorgesen and colleagues [37] reported that life satisfaction, the subjective judgment on the life situation, seems to be a low level soon after the injury, then increase to a stable level over longer period, even if it is lower than general population. Some authors described differences between younger and older people with spinal cord injury and more studies are needed to better comprehend the data. Frontera and Mollett [2] reported decline in satisfaction in different dominions of life (like social and sexual life, and general health) but an increased satisfaction in other dominions (like financial, living situations and employment). They also claimed that employment and personal independence seem to have a positive effect of satisfaction in aging people [2].

## 4. Discussion and Conclusions

To summarize, the present paper aimed to discuss some issues from recent literature on the relationship between aging and disability in a specific disabling condition, the spinal cord injury, according to a narrative review approach. According to the data resulting from the selected articles, the relationship between aging and spinal cord injury is a very complex one.

As in Frontera and Mollett [2], we can summarize some points of interest in the study of the relationship between aging and spinal cord injury:

1. From a prevention point of view, the progressive increasing of the mean age of lesion is related to the increase of some specific causes of lesions that can be prevented (falls and other kind of domestic/road accidents);
2. From a treatment point of view, the awareness that people with spinal cord injury could have increased life expectancy after the lesion but they could also have an higher number of secondary health complications after lesions, puts attention on the need of specific care to specific secondary health complications and to comorbidities;
3. Again, from a treatment point of view, the risk of an “accelerated ageing” in people with spinal cord injury need to be taken into account in short, medium and long-term interventions, aiming to control the process and to limits its consequences.
4. The increased awareness of the psychological and psychosocial consequences of spinal cord injury and their features in acute, post-acute state and in other phases of life after the lesion puts attention on the need of specific psychological and psychosocial interventions, during the life of people with spinal cord injury.
5. The awareness that disability is a process (and it is related to the consequences of the relationship between health condition and environment) needs to be taken into account to better study this relationship and to better study factors that promote functioning, participation and better quality of life of people with spinal cord lesion.

In general, the findings in this field highlighted the need for a most comprehensive approach, based on a better awareness of acute needs, short-term needs and long-term needs of people aging with spinal cord injury and the need on an integrated approach, based on a biopsychosocial vision of spinal cord lesion. They also highlight the need of a transdisciplinary approach in this field, aiming to taking into account all the life domains of people with spinal cord injury, and the need of a close link between the different professionals that have a role in the care and in the rehabilitation of people with spinal cord injury. Even if we have now more information than in the past, more studies are needed to better comprehend the complex relationship between aging and spinal cord injury and their effects on quality of life of people. The aims of this studies is to help professionals to formulate guidelines for treatments, rehabilitation and community care to promote health, to prevent disease and secondary health conditions, to support wellbeing and good quality of life and to postpone functional decline [2, 33, 42–44].
